# Modified Adenosines Sensitize Glioblastoma Cells to Temozolomide by Affecting DNA Methyltransferases

**DOI:** 10.3389/fphar.2022.815646

**Published:** 2022-04-26

**Authors:** Maria Chiara Proto, Donatella Fiore, Chiara Piscopo, Chiara Laezza, Maurizio Bifulco, Patrizia Gazzerro

**Affiliations:** ^1^ Department of Pharmacy, University of Salerno, Fisciano, Italy; ^2^ Institute of Endocrinology and Experimental Oncology, IEOS CNR, Naples, Italy; ^3^ Department of Molecular Medicine and Medical Biotechnologies, University of Naples “Federico II”, Naples, Italy

**Keywords:** glioblastoma, N6-isopentenyladenosine, N6-benzyladenosine, temozolomide, chemoresistance, MGMT, Fbxw7, DNA methyltransferases

## Abstract

Glioblastoma (GBM) is the most common and lethal primary malignant brain tumor, and due to its unique features, its management is certainly one of the most challenging ones among all cancers. N6-isopentenyladenosine (IPA) and its analog N6-benzyladenosine (N6-BA) are modified nucleosides endowed with potent antitumor activity on different types of human cancers, including GBM. Corroborating our previous finding, we demonstrated that IPA and N6-BA affect GBM cell line proliferation by modulating the expression of the F-box WD repeat domain-containing-7 (FBXW7), a tumor suppressor with a crucial role in the turnover of many proteins, such as SREBPs and Mcl1, involved in malignant progression and chemoresistance. Luciferase assay revealed that IPA-mediated upregulation of FBXW7 translates in transcriptional inactivation of its oncogenic substrates (Myc, NFkB, or HIF-1α). Moreover, downregulating MGMT expression, IPA strongly enhances the killing effect of temozolomide (TMZ), producing a favorable sensitizing effect starting from a concentration range much lower than TMZ EC50. Through DNA methyltransferase (DNMT) activity assay, analysis of the global DNA methylation, and the histone modification profiles, we demonstrated that the modified adenosines behave similar to 5-AZA-dC, known DNMT inhibitor. Overall, our results provide new perspectives for the first time, suggesting the modified adenosines as epigenetic tools able to improve chemo- and radiotherapy efficacy in glioblastoma and potentially other cancers.

## Introduction

Gliomas comprise a disparate group of brain tumors with distinct biological and clinical properties. Glioblastoma multiforme (GBM) is the most aggressive grade IV subtype, with poor prognosis and a median survival of about 15 months after surgery and subsequent treatment with radiations and chemotherapy. Despite the efforts to identify new efficacious antitumor agents that are able to cross the blood–brain barrier, to date, temozolomide (TMZ) remains the most used drug in GBM treatment, but unfortunately, the onset of chemoresistance results in treatment failure and tumor recurrence (Stupp et al., 2005; Goenka et al., 2021). The main mechanism of TMZ resistance is related to the overexpression of the DNA repair protein O6-methylguanine DNA methyltransferase (MGMT), which neutralizes the alkylating effect of the drug. As a matter of fact, MGMT promoter methylation is considered a favorable prognostic biomarker of TMZ sensitivity (Oldrini et al., 2020). However, several factors contribute to GBM malignancy and tumor recurrence, including heterogeneity, hypermutation, immune-suppressive tumor microenvironment, and, last but not least, epigenetic landscape (Goenka et al., 2021; Wu et al., 2021). In the last decades, epigenetic aberrations in GBM became an attractive topic for the identification and development of new therapeutic strategies since DNA methylation, histone modifications, and chromatin remodeling are widely involved in tumorigenesis and tumor-related mechanisms. Of note, the need to identify new therapeutic targets has led to considering many epidrugs in the treatment of GBM, with promising results (Wu et al., 2021).

Modified adenosines, such as N6-benzyladenosine (N6-BA) and, in particular, N6-isopentenyladenosine (IPA), have received increasing attention as antitumor agents in several cancers. The IPA*-*induced antiproliferative effect *in vitro* was related to the inhibition of the farnesyl diphosphate synthase (FDPS), a key enzyme of the mevalonate pathway crucial for cholesterol biosynthesis and prenylation of oncogenic proteins, mainly belonging to the RAS super-family (Laezza et al., 2006; Abate et al., 2017). Moreover, supporting previous observations, a structural interaction between IPA and FDPS has been shown *in silico* (Scrima et al., 2014). However, the multitude of their effects described in numerous studies (Laezza et al., 2009; Ottria et al., 2010; Ciaglia et al., 2014; Ciaglia et al., 2017a; Ciaglia et al., 2017b; Dassano et al., 2014; Pisanti et al., 2014; Ciaglia et al., 2018; Ranieri et al., 2018), and the chemical nature of these molecules, suggests that a precise mechanism explaining their pleiotropic effects in different tumor-related mechanisms, ranging from the tumor microenvironment to immunomodulation, has not yet been fully identified. Recently, we reported that IPA inhibits colorectal cancer (CRC) proliferation *in vitro* and *in vivo* by increasing the expression of the tumor suppressor FBXW7 and, in turn, regulating the FBXW7/SREBP/FDPS axis, corroborating with previous studies (Fiore et al., 2019). FBXW7 is a component of the SCF-E3 ubiquitin ligase complex responsible for the degradation of various oncoproteins and transcription factors, such as p100/NFκB, HIF-1α, c-Myc, SREBP, and Mcl1 (Sailo et al., 2019). Mutation, deletion, and promoter hypermethylation are the main events causing FBXW7 inactivation and consequent imbalance of its oncogenic substrates, thereby leading to tumor progression in many types of human cancers. p53 mutations, reported to be associated with DNA methyltransferase 1 (DNMT1) protein overexpression, are also associated with FBXW7 promoter hypermethylation, resulting in decreased FBXW7 transcript levels (Kitade et al., 2016). In the brain, FBXW7 helps the neural stem cell differentiation, and its abrogation may result in increased self-renewal ability because of the deregulation of Notch, c-Jun, and c-Myc (Takeishi and Nakayama, 2014). Recently, clinical tissues and TCGA (The Cancer Genome Atlas) database analysis revealed that FBXW7 expression was inversely correlated with glioma histology and positively with patient survival time. Moreover, *in vitro* FBXW7 overexpression significantly suppressed proliferation, invasion, and migration and, most importantly, was found to increase temozolomide (TMZ) toxicity in a resistant clone (Lin et al., 2018). We previously found that, in *FBXW7*- and *TP53*-wild-type CRC cells, IPA-mediated restoration of FBXW7 tumor suppressor translates into synergism with 5-fluorouracil (Fiore et al., 2019).

In this work, we extended the analysis of IPA and its synthetic analog N6-BA effects on the FBXW7/SREBP/FDPS axis in GBM models. Interestingly, we demonstrated for the first time that the modified adenosines behave like epidrugs, modulating the expression and activity of DNMTs and that IPA enhances the effect of TMZ in MGMT-methylated cells.

## Materials and Methods

### Reagents and Antibodies

N6-isopentenyladenosine (IPA), N6-benzyladenosine (N6-BA), 5-fluorouracil (5-FU), temozolomide (TMZ), and decitabine (5-Aza-2′-deoxycytidine or 5-AZA-dC) were purchased from Sigma-Aldrich (St. Louis, MO, United States) and dissolved in sterile DMSO.

The following primary antibodies were purchased from Cell Signaling Technology (Beverly, MA, United States): anti-GAPDH monoclonal; anti-Mcl-1; and anti-Phospho-Histone H2Ax (Ser139); anti-SREBP-1 was purchased from Santa Cruz Biotechnology (Dallas, TX, United States). The following primary antibodies were purchased from Abcam (Cambridge, United Kingdom): anti-FBXW7; anti-FDPS; anti-MGMT; anti-DNMT1; anti-histone H3, H3K27me3, and H3Ac (pan-acetyl K9 + K14 + K18 + K23 + K27). Anti-phospho-c-Myc (Thr58) was purchased from Thermo Fisher Scientific (Waltham, MA, United States). The goat anti-rabbit secondary antibody and goat anti-mouse secondary antibody were purchased from Abcam.

### Cell Cultures

Human GBM cell lines U87MG (U87), U251MG (U251), and T98G (T98) were purchased from Cell Lines Service GmbH (Eppelheim, Germany) and cultured in Eagle’s Minimal Essential Medium (EMEM) with 10% FBS, 2 mM L-glutamine, 50 ng/ml streptomycin, 50 units/mL penicillin, 1% non-essential amino acids, and 1 mM sodium pyruvate.

GBM primary cell lines were established as previously described (Ciaglia et al., 2017a). In brief, small pieces of surgical brain tissues containing tumor were collected at the time of craniotomy at the Neurosurgery Service of “G. Rummo” Medical Hospital (Benevento, Italy), divided into portions stored at −80°C for subsequent molecular characterization (RNA, DNA, and protein extraction), or immediately processed to generate primary tumor cell lines. A second sample from each patient was also taken for clinical diagnosis performed by expert neuropathologists in accordance with the International Classification of CNS tumors drafted under the auspices of the World Health Organization (WHO). The tumors were diagnosed as astrocytoma (WHO grade I–III), glioma (WHO grade II), or glioblastoma multiforme (WHO grade IV). All tissue samples were collected in accordance with the ethical standards of the Institutional Committee. The patients had been informed about the establishment of cellular models from their tumors and had given informed consent in written form. The preparation of adherent primary cultures of brain tumor cells was conducted through Miltenyi technology using the Brain Tumor Dissociation Kit (Miltenyi Biotec, Calderara di Reno, Italy) and gentleMACS, combining enzymatic and mechanic dissociation. GBM primary cell lines (designated ad GBMn) were kept in culture in DMEM/F12 supplemented with 15% heat-inactivated fetal bovine serum (Euroclone), 2% L-glutamine, 1% antibiotic mixture, 1% sodium pyruvate, and 1% non-essential amino acids (Euroclone). They were routinely grown in monolayers and maintained at 37°C in a 5% CO2 humified atmosphere and regularly tested for mycoplasma presence. Experiments were performed using passages II–VI of these cells.

### Cell Proliferation and Viability Assay

Cell proliferation was evaluated through a colorimetric ELISA kit based on 5-bromo-2′-deoxy-uridine (BrdU) labeling and detection (Roche Diagnostics GmbH, Mannheim, Germany). In brief, CRC or GBM cells were seeded into 96-well plates at a density of 5–8×10^3^ cells/well and treated in triplicate with IPA, N6-BA (ranging from 0.5 to 20 µM), or vehicle (DMSO) as a control for 24 or 48 h. At the end of incubation time, the medium was removed and cells were fixed/denatured for 30 min. Then, cells were first incubated with an anti-BrdU peroxidase-conjugated antibody solution (anti-BrdU-POD) for 90 min and then with a substrate solution for about 20 min. The colorimetric reaction was monitored and measured through a microplate reader (Multiskan™ GO Microplate Spectrophotometer, Thermo Scientific) at 370 nm. The blank was performed in each experimental setup. The absorbance value of the blank was subtracted from other experimental values, and cell proliferation was expressed as the percentage of absorbance values ±SD of treated samples to untreated controls of three separate experiments in triplicate.

To evaluate the effect of IPA pre-treatment on TMZ toxicity, GBM cells were seeded into 96-well plates at a density of 5 × 10^3^ cells/well and exposed to 10 µM IPA or vehicle alone for 24 h. After the incubation, the IPA-containing medium was removed and replaced with fresh medium added with 5, 50, 250, and 500 µM TMZ or the relative vehicle alone for 72 h. Cell viability was evaluated through a colorimetric MTT metabolic activity assay. To this aim, MTT stock solution (5 mg/ml in PBS, Sigma) was added to each well and incubated for 4 h at 37°C in humidified CO2. At the end of the incubation, the medium was removed, and the formazan crystals were solubilized with 100 μL of DMSO. MTT conversion to formazan by metabolically viable cells was monitored by using a spectrophotometer at an optical density of 540 nm. Each data point represents the average of at least three separate experiments in triplicate.

### Western Blot Analysis

Total protein extracts were obtained lysing cultured cells with ice-cold RIPA buffer (50 mM Tris-HCl pH 8.0, 150 mM NaCl, and 1% Nonidet P-40) supplemented with protease and phosphatase inhibitors (Sigma). Cellular extracts optimized to preserve DNMT enzymatic activity assay were obtained through the EpiQuik™ Nuclear Extraction Kit (EpiGentek Group Inc, NY, United States) following the manufacturer’s instructions. Purification of total histones was obtained according to the protocol provided by the Histone Extraction Kit (Abcam). Protein concentration was determined through the Bradford method, and samples were subjected to 10–12% SDS-PAGE. Gels were electroblotted into nitrocellulose membranes that were probed with the primary antibodies described above. Membranes were incubated with enhanced chemiluminescence (ECL) reagent solution (GE Healthcare, Hilden, Germany) and exposed to X-ray film (Santa Cruz). Immunoreactive band density was quantified using ImageLab v4.0 analysis software (Bio-Rad, Hercules, CA, United States).

### Luciferase Assay

Cells were transiently co-transfected with a firefly luciferase construct (100 ng) containing the transcriptional responsive elements (TREs) of selected genes and the Renilla luciferase vector (10 ng) to normalize transfection efficiency (Cignal Finder Reporter assay kit, QIAGEN, Hilden, Germany). A non-inducible reporter construct encoding firefly luciferase under the control of a basal promoter element (TATA box), without any additional TREs, was used as a negative control. The efficiency of transfection was evaluated in cells transfected with a GFP construct as a reporter. After 18 h from transfection, a dual-luciferase assay was performed according to the manufacturer’s instruction (Promega, Madison, WI, United States). Luciferase readings were measured using an EnSpire-2300 luminometer (Perkin Elmer, Waltham, MA, United States). Data were represented as relative luciferase activity, obtained by the ratio of firefly values (promoter reporter) to Renilla values (control reporter). Experiments in triplicate were repeated at least three times, and values were expressed as the mean ± SD.

### RNA Extraction and PCR

Total RNA extraction, cDNA synthesis, and reverse-transcription PCR were performed as previously described (Proto et al., 2012). Primer pairs specific to human FDPS (5′-CAG​ATC​TGC​TGG​TAT​CAG​AA-3′ forward and 5′-GTG​CTC​CTT​CTC​GCC​ATC​AAT-3′ reverse), human MGMT (5′-CCT​GGC​TGA​ATG​CCT​ATT​TC-3′ forward and 5′-GCT​GCT​AAT​TGC​TGG​TAA​G-3′ reverse), human actin B (5′-ACT​GGG​ACG​ACA​TGG​AGA​A-3′ forward and 5′-ATC​TTC​ATG​AGG​TAG​TCA​GTC​A-3′ reverse), or human β2-microglobulin (5′-CCT​GGA​TTG​CTA​TGT​GTC​TGG​GTT​TCA​TCC-3′ forward 5′-GGA​GCA​ACC​TGC​TCA​GAT​ACA​TCA​AAC​ATG-3′ reverse) were used. All reactions were performed at least in triplicate in three independent experiments; the PCR products were quantified using ImageLab v4.0 analysis software (Bio-Rad), and results were normalized to those obtained from actin B or β2-microglobulin.

### Genomic DNA Extraction

U87, U251, and T98 cells were plated into 60 mm dishes, treated with 10 µM IPA, 10 µM N6-BA, 500 µM 5-AZA-dC, or IPA-5-AZA-dC, and collected after 24 h. Pellets of about 1 × 10^6^ cells were dissolved overnight in saline tris-EDTA (1X STE Buffer, 10 mM Tris-HCl pH 8, 1 mM EDTA, and 100 mM NaCl) solution containing 0.5% sodium dodecyl sulfate and 0.2 mg/ml proteinase K (Sigma) at 55°C with shaking. After overnight incubation, phenol/chloroform extraction was performed to denature and remove the protein content. In brief, the solution was mixed with phenol:chloroform:isoamyl alcohol (25:24:1 in volume) solution (Sigma) for 10 min and centrifuged at 5,000 rpm for 20 min at room temperature. The upper clear aqueous phase containing DNA was collected into a new tube, precipitated with 1/10 volume of 3M sodium acetate (pH 5.2) and 2,2 volume of cold absolute ethanol, and then, kept at −80°C for at least one hour. After washing with cold 70% ethanol, the pelleted DNA was air-dried, dissolved in 1X TE (1 mM Tris-HCl pH 8, 0.1 mM EDTA), and quantified with NanoDrop 2000 (Thermo Fisher Scientific).

### DNMT Activity Assay

GBM cell lines were treated for 24 h with 10 µM IPA or N6-BA, 5-Aza-2′-deoxycytidine (5-AZA-dC or decitabine), or a combination of IPA and 5-Aza-dC. To obtain the inhibition of DNMTs, the cells were treated with 5-AZA-dC 500 µM for 24 h, in order to adapt the treatment to the experimental conditions necessary for the effect of IPA or N6-BA without overt cytotoxicity. Nuclear extracts containing cell-derived DNMT enzymes were obtained through the EpiQuik™ Nuclear Extraction kit (EpiGentek Group Inc, NY, United States). DNMT enzymatic activity (*de novo*, maintenance) was measured using the DNMT Activity Assay Kit (ab113467, Abcam), a colorimetric kit based on ELISA-like reaction, following the manufacturer’s instructions. In brief, optimized nuclear extracts (10 µg) containing purified DNMTs, positive and negative control, were diluted with 1X AdoMet working buffer and incubated at 37°C for 120 min, in microplate wells coated with DNA substrate. Subsequently, the wells were washed three times with 1X Wash Buffer and then incubated with the diluted capture antibody for 60 min, at room temperature protected from the light. After washing, the plate was incubated with diluted detection antibody and then with diluted enhancer solution for 30 min. Finally, for signal detection, developer solution was added, and the colorimetric reaction was monitored for 10 min, away from direct light, until development of blue color was detected in positive control wells. The reaction was stopped by adding stop solution, and the absorbance was immediately read at 450 nm and 655 nm for wavelength correction. The ratio which is proportional to enzyme activity was calculated using the formula

$$\text{DNMT}\;\text{activity}\left(\text{OD}/\text{h}/\text{mg}\right)=\frac{\text{Sample}\;\text{OD}-\text{blank}\;\text{OD}}{\text{Protein}\;\text{amount}\left(\text{µg}\right)\times \text{hour}\left(\text{h}\right)}\times 1000.$$

### Global DNA Methylation Assay

Global DNA methylation was evaluated through the MethylFlash™ Global DNA Methylation (5-mC) ELISA Easy Kit (EpiGentek Group Inc, NY, United States) measuring levels of 5-methylcytosine (5-mC). About 100 ng of input DNA from GBM cells, extracted using the previously described protocol, was added to a microplate well with a high affinity for DNA binding. Negative control (NC) and different percentages of positive control (PC) were prepared according to the manufacturer’s indications to set the standard curve and determine the slope. The plate was covered and incubated at 37°C for 60 min. Following washing, 5-mC detection complex solution was added to the plate and removed after 50 min at room temperature. Colorimetric reaction was triggered by the addition of Developer Solution and stopped when the 5% PC turned medium blue. Absorbance was immediately read at 450 nm. DNA methylation was calculated as follows:

$$5\text{mC\%}=\frac{\text{Sample}\;\text{OD}-\text{NC}\;\text{OD}}{\text{Slope}\times \text{input}\;\text{DNA}\;\text{amount}\left(\text{ng}\right)}\times 100.$$

### Statistical Analysis

Data obtained from multiple experiments were calculated as means ± SD, if not otherwise specified, and analyzed for statistical significance by using the two-tailed Student t-test. All data shown are representative of at least three independent experiments performed in triplicate. Values of *p* < 0.05 were considered statistically significant.

## Results

### IPA and N6-BA Affect GBM Cell Proliferation by Increasing FBXW7 Expression

It was previously reported that IPA and N6-BA exerted a marked cytostatic activity against the glioma cell line U87 but did not affect normal human astrocytes viability. The antiproliferative effect in GBM was mainly associated with the induction of apoptosis, along with blockade of FDPS-dependent protein prenylation, which counteracted EGF receptor-mediated oncogenic signaling (Ciaglia et al., 2017a, Ciaglia et al., 2017b). Glioma proliferation is significantly impaired when FBXW7 is overexpressed *in vitro*, suggesting its tumor suppressive role in astroglial cells (Hagedorn et al., 2007). In turn, FBXW7 downregulation by inactivating mutations or silencing is frequently correlated with G-IV tumors (Sailo et al., 2019). With the aim to corroborate our results in CRC (Fiore et al., 2019) and extend the study to other modified adenosines and investigate if FBXW7 modulation is a common mechanism in other cancer systems, we evaluated IPA and N6-BA effects in GBM models (U87, U251 and T98) exhibiting different FBXW7 basal levels. We first assessed cell proliferation and confirmed that, from 5 μM, IPA and N6-BA reduced the proliferation of U87 by over 40%. In U251 cells, a strong, dose-dependent inhibitory effect was observed starting from 24 h of treatment with both IPA and N6-BA at the lowest concentration. A significant but fair reduction of proliferation was only detected at the highest dose of IPA or from 5 µM N6-BA after 48 h in the T98 cell line (Supplementary Figure S1).

**FIGURE 1:**
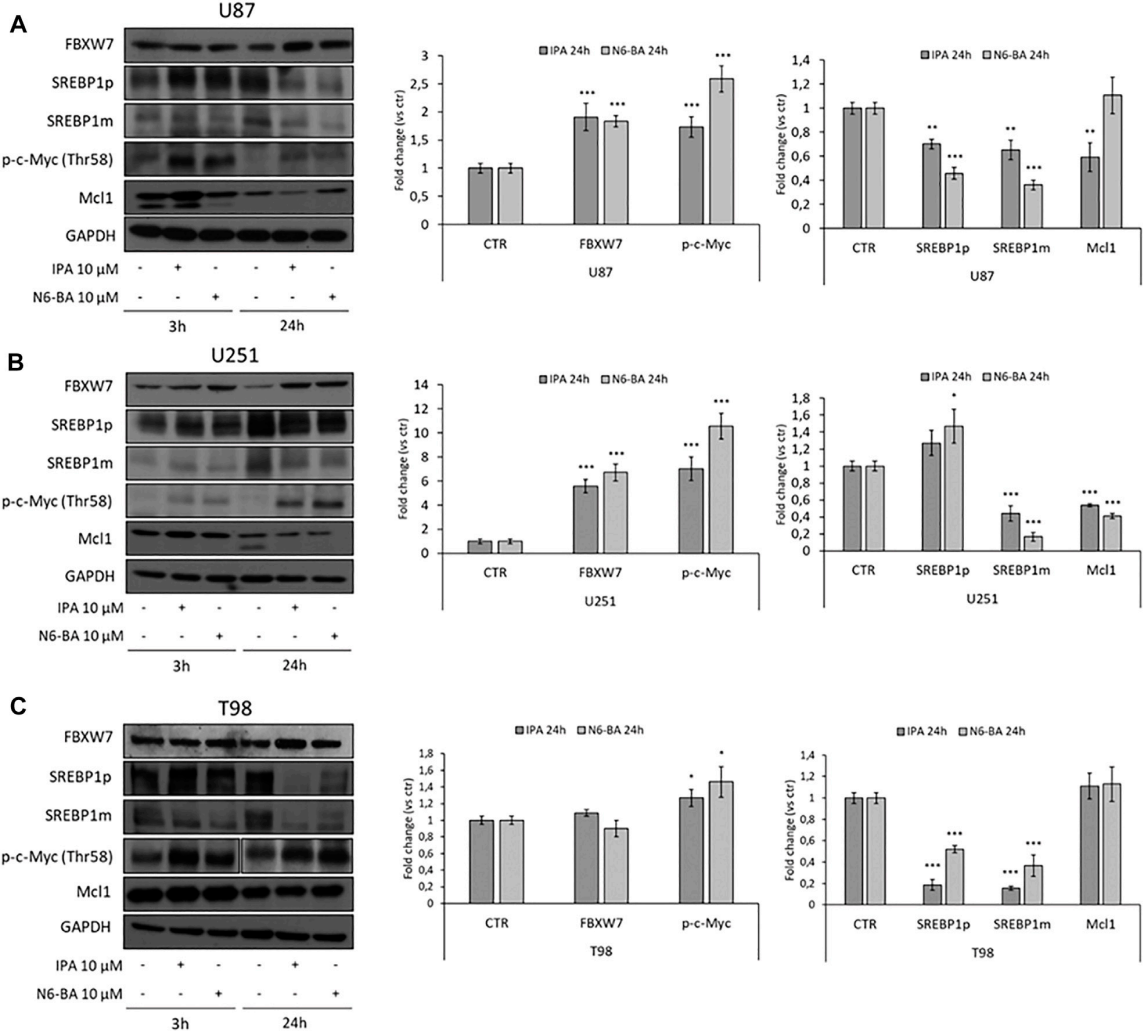
IPA and N6-BA effect on FBXW7 modulation in GBM cells. Representative western blot of FBXW7, SREBP1 precursor (SREBP1p) and mature form (SREBP1m), phosphorylated c-Myc (Thr58) and Mcl1 protein expression in U87 **(A)**, U251 **(B),** and T98 **(C)** cells treated for 3 and 24 h with IPA or N6-BA. The histograms on the right represent the relative densitometric analysis at 24 h. GAPDH was used as a loading control. Data are expressed as mean ± SD of at least three independent experiments. *p < 0.05, **p < 0.01, and ***p < 0.005 vs. control.

Next, we analyzed the expression of FBXW7 and its targets in our GBM *in vitro* models. In U87 and U251 cell lines, in which FBXW7 expression is suppressed because of promoter hypermethylation (Gu et al., 2007) and both are particularly sensitive to the molecules, 10 µM IPA and N6-BA strongly upregulated FBXW7 after 24 h. As observed in CRC models (Fiore et al., 2019), the examined downstream targets were coherently modulated with a trend reflecting that of FBXW7. Phosphorylation on c-Myc residue Thr58, a tag of FBXW7-dependent degradation, increased at early time was sustained and highly statistically significant after 24 h, especially in U251 where the treatments augmented FBXW7 expression over 4-fold. Similarly, active SREBP1 and Mcl1 levels were also reduced (Figures 1A,B). Under the same experimental conditions, in FBXW7-wild-type T98 cells, IPA and N6-BA failed to significantly inhibit cell proliferation and modulate FBXW7 protein levels. However, coherent with the constitutive and normal expression of FBXW7, in this cell line, both the compounds were able to increase Thr58 c-Myc phosphorylation and efficiently reduce SREBP1 precursor and active form (Figure 1C).

### IPA Affects the Transcriptional Activity of FBXW7 Substrates

**FIGURE 2:**
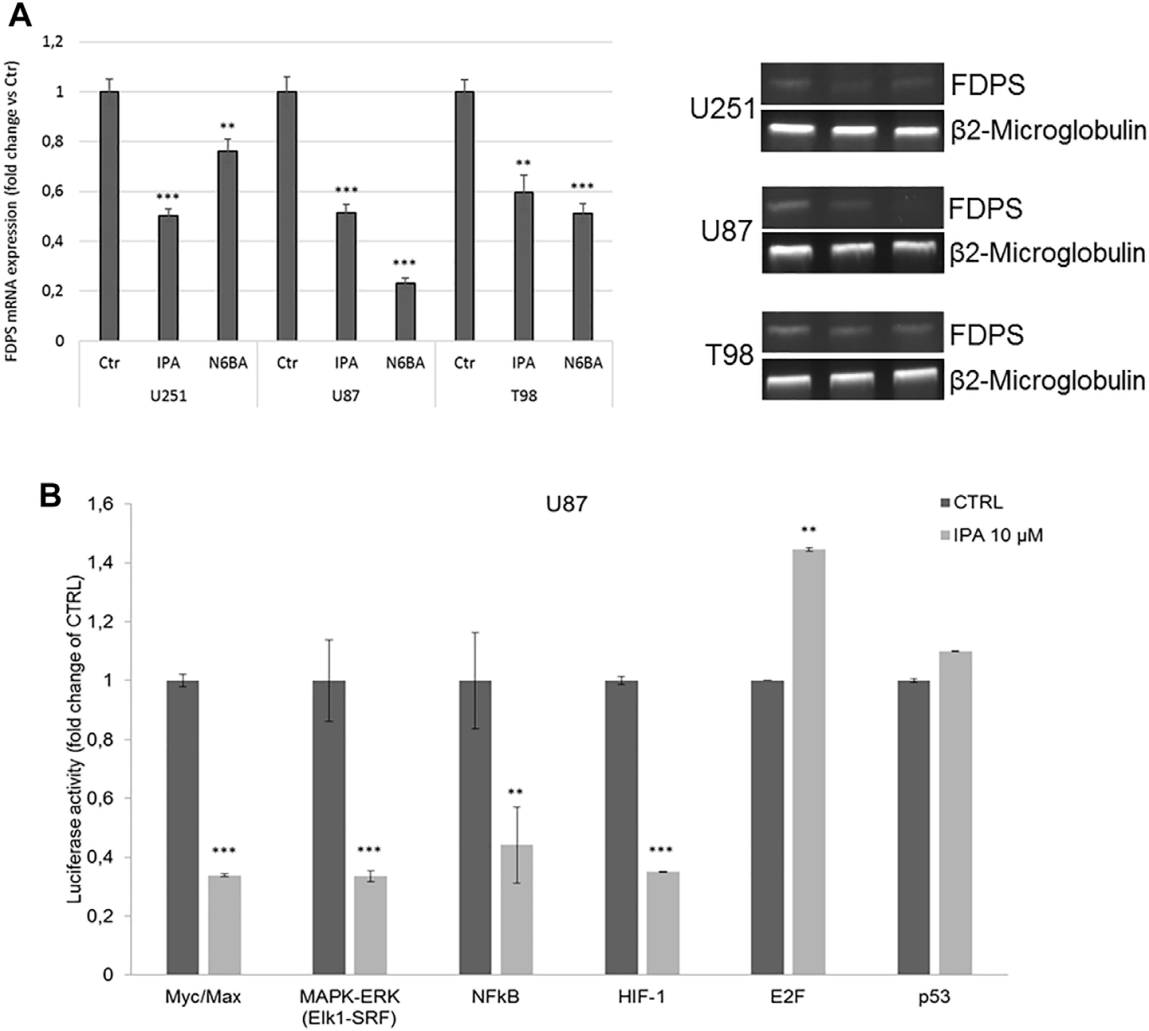
IPA affects the transcriptional activity of FBXW7 substrates. **(A)** Representative FDPS mRNA expression (left panel) and densitometric analysis (right panel) in U251. U87 and T98 cells were treated with IPA 10 µM or N6-BA 10 μM for 24 h. Data are expressed as mean ± SD of at least three independent experiments. **p < 0.01, ***p < 0.005 vs. control. β2-Microglobulin was used as a reference gene. **(B)** Luciferase activity on selected cancer-related Transcriptional Responsive Elements (TREs). Analysis of luciferase activity of TREs controlled by Myc, ERK, NFkB, HIF-1α, E2F, or p53 in the U87 cell line. The histograms represent luciferase activity measured at 18 h from transfection with a reporter construct containing the TRE elements and treated with IPA 10 μM or vehicle. Firefly luciferase was normalized to Renilla luciferase reading, and the data were plotted as fold change (mean ± SD of four independent experiments in triplicate; unpaired two-tailed Student’s t-test; **p < 0.01; ***p < 0.005) compared to control cells.

IPA and N6-BA impact c-Myc protein stabilization and reduce SREBP1 levels, especially in U87 and U251 cells, where they strongly re-express FBXW7, even in T98. Analysis of FDPS mRNA expression confirms the ability of IPA and N6-BA to interfere with the SREBP1/FDPS axis, reducing FDPS expression (Figure 2A). This evidence suggests that the molecules could potentially influence GBM dynamics through transcriptional mechanisms in an FBXW7-dependent or -independent way. To further corroborate our results, we examined the luciferase activity on the Transcriptional Regulatory Elements (TREs) of c-Myc and additional selected effectors playing a crucial role in cancer progression. In IPA-treated U87 cells, the luciferase activity on Myc/Max E-box was significantly reduced, suggesting that the IPA-mediated increase of c-Myc Thr58 phosphorylation effectively represents a tag for its degradation and consequent inactivation. Furthermore, the luciferase assay also revealed a strong downregulation of other oncogenic pathways, including MAPK/ERK, as expected due to the IPA-induced EGFR degradation (Ciaglia et al., 2017a), NFkB and HIF-1, both FBXW7 substrates that together cooperate to inflammation and oxidative stress response. Notably, their transcriptional inactivation aligns with the induction of NRF2 and the reported anti-inflammatory and anti-angiogenic IPA activity (Ciaglia et al., 2014; Dassano et al., 2014; Pisanti et al., 2014). Coherently, the suppression of cancer survival pathways corresponded to an increase of Rb/E2F and, despite not significantly, of p53 transcriptional activity, matching to the U87 proliferation arrest (Figure 2B).

### IPA Could Sensitize GBM Cells to Temozolomide

**FIGURE 3:**
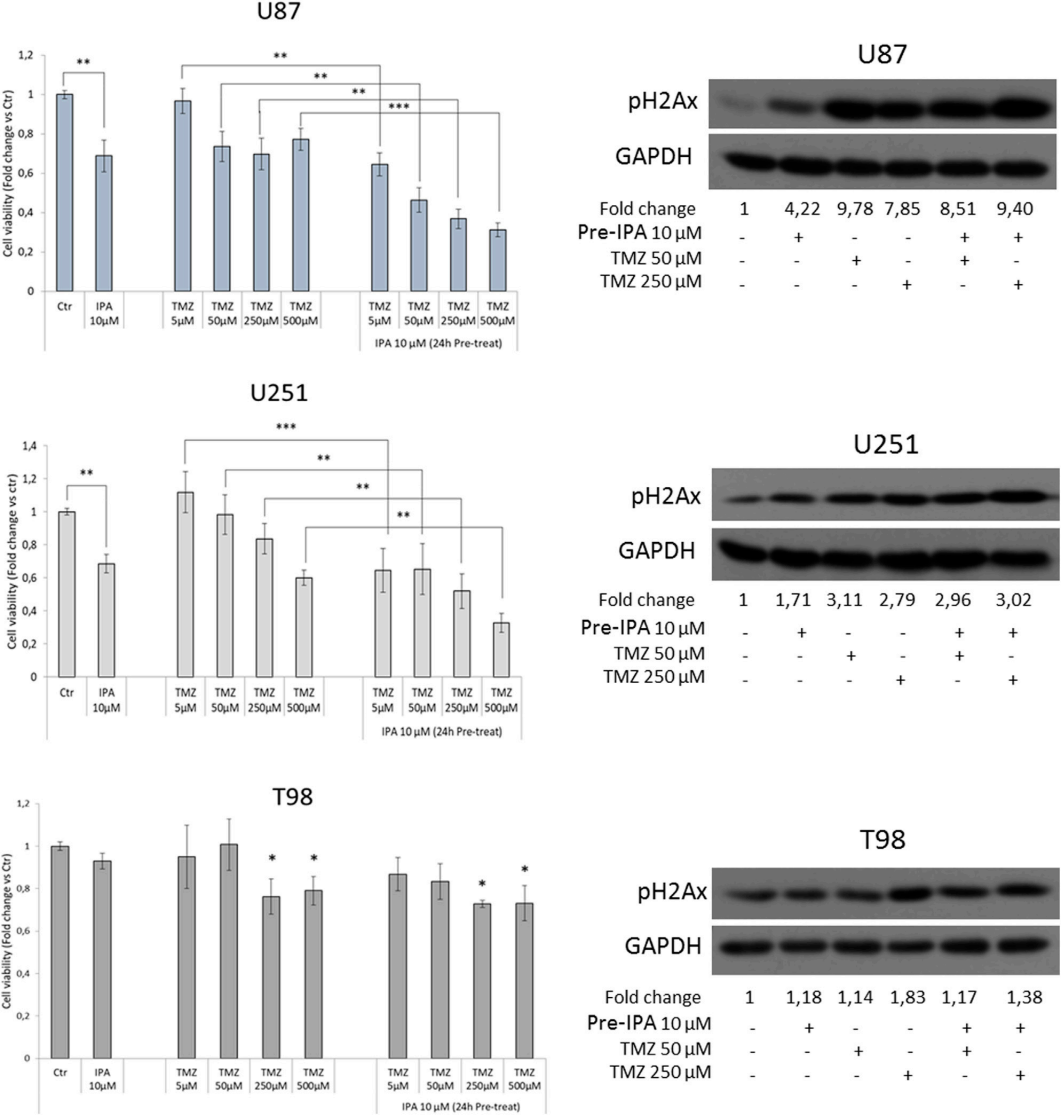
IPA sensitizes GBM to TMZ. GBM cells were exposed to IPA 10 µM or vehicle (DMSO) for 24 h. After the incubation, the medium was removed and replaced with a fresh one containing the indicated concentrations of TMZ or the relative vehicle alone. MTT assay was used to assess cell viability after 72 h, expressed as the mean ± SD of three independent experiments. **p < 0.01, ***p < 0.005 in U87 and U251, *p < 0.05 vs. control in T98 (histograms on the left). On the right, representative western blot and relative densitometric analysis (fold change vs. control) of phosphorylated histone H2Ax (S139), in U87, U251, and T98 cells subjected to the same experimental conditions of the vitality assay (panel on the left).

Our research group recently showed that IPA might act as a radio-sensitizing agent in GBM by attenuating RAD51 foci formation, thus increasing DNA damage before irradiation (Navarra et al., 2020). Because of its involvement in DNA double-strand breaks, FBXW7 was correlated with GBM resistance to ionizing radiation (Humphreys et al., 2021). Moreover, a combination of FBXW7 overexpression and temozolomide (TMZ) treatment was reported to notably sensitize U251 cells to the cytotoxic effect of the drug (Lin et al., 2018). Based on this evidence, to further explore the role of the molecules in cancer resistance mechanisms, we evaluated the IPA effect in sensitizing GBM to TMZ *in vitro*. To this aim, GBM cells were exposed to IPA 10 µM for 24 h. After the incubation, the medium was removed, and cells were treated for an additional 72 h with TMZ (5–50–250–500 µM). In the TMZ sensitive U87 and U251 cells, IPA pre-treatment was able to significantly ameliorate the killing effect of TMZ at all the used concentrations and in a dose-dependent manner. This result was further confirmed by the analysis of H2Ax phosphorylation levels, a mark of DNA damage, induced after 72 h by TMZ alone (50 or 250 µM) and incremented with IPA pre-treatment (Figure 3). In TMZ-resistant T98 cells, despite TMZ produced a weak significant reduction of cell viability at the highest concentrations, IPA pre-treatment did not improve its effect at any concentrations tested. Coherently pH2Ax levels were significantly affected only with TMZ 250 µM (Figure 3).

Similar to IPA, the N6-BA analog sensitizes GBM cells to TMZ. In particular, N6-BA produces the strongest effect in the TMZ-responsive U87 and U251 cells, starting from 5 to 50 µM of TMZ, respectively. Similar to IPA, N6-BA also failed to sensitize TMZ-resistant T98 cells (Supplementary Figure S2).

**FIGURE 4:**
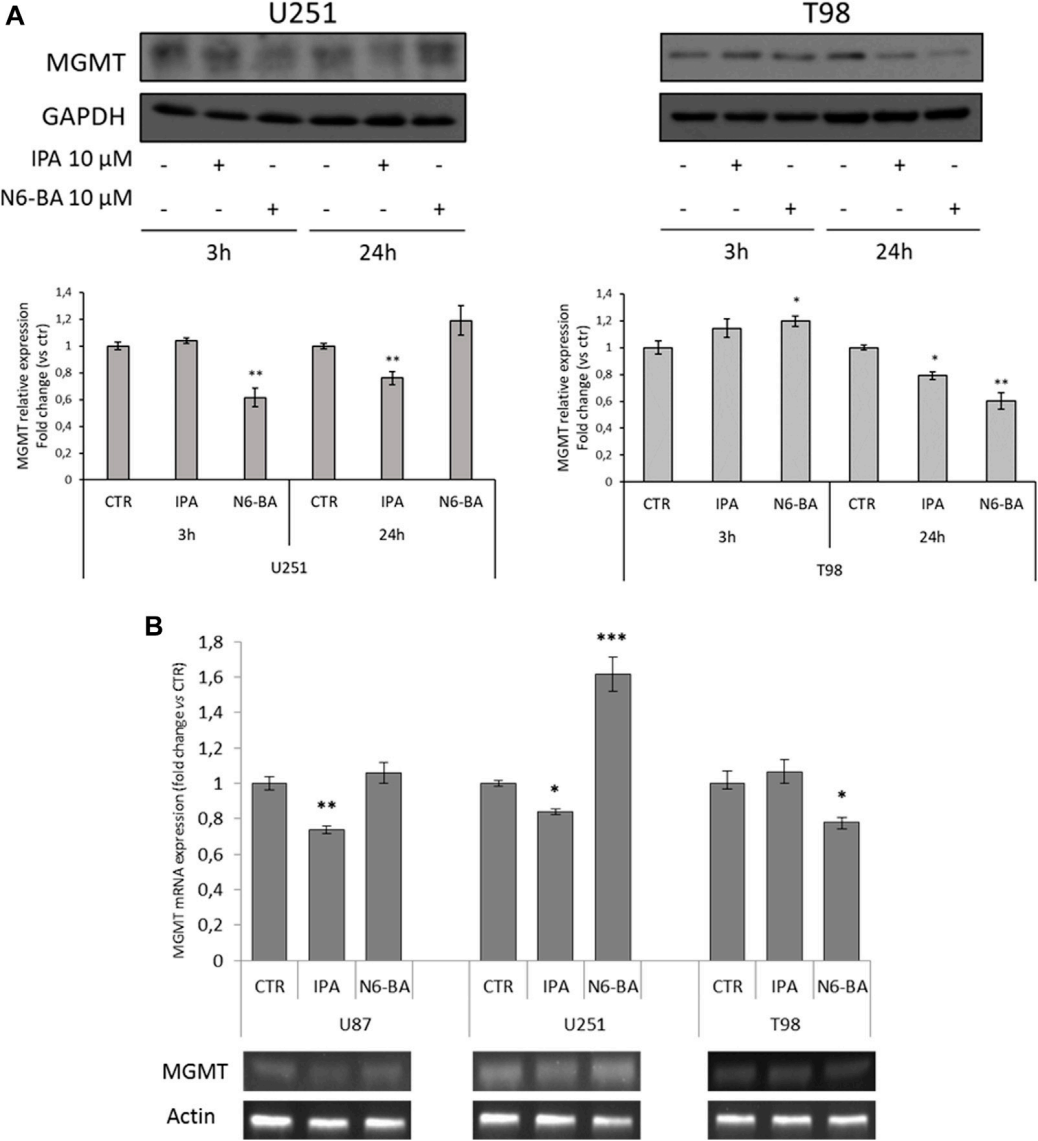
IPA and N6-BA modulate MGMT protein levels. **(A)** Representative western blot and densitometric analysis of MGMT protein expression in U251 and T98 cells treated with 10 µM IPA or 10 µM N6-BA. GAPDH was used as a loading control. **(B)** Representative MGMT mRNA expression (lower panel) and densitometric analysis (upper panel) in U87, U251, and T98 cells treated with 10 µM IPA or 10 µM N6-BA, for 24 h. Data are expressed as mean ± SD of at least three independent experiments. *p < 0.05, **p < 0.01, and ***p < 0.005 vs. control.

As we mentioned before, in GBM, TMZ resistance is primarily due to the overexpression of MGMT, which neutralizes the alkylating effect of the drug by removing methyl groups from DNA. Indeed, epigenetic inactivation of the MGMT gene is commonly accepted as a favorable prognostic biomarker of TMZ sensitivity (Oldrini et al., 2020). We then analyzed MGMT protein levels in U251 and T98, respectively, characterized by constitutively low and high expression levels of the enzyme and different sensitivity to TMZ (Lee, 2016). In U251, known to have a high percentage of CpG methylation in the MGMT promoter, with different trends, both IPA and N6-BA were able to further reduce MGMT expression. More interestingly, MGMT levels were efficaciously reduced after 24 h also in T98, TMZ-resistant cell line that has shown to be less sensitive to the molecules (Figure 4A). Analysis of mRNA expression (Figure 4B) revealed that in both hypermethylated cell lines, U87 and U251, IPA was able to further reduce MGMT mRNA levels after 24 h of treatment. In U251, coherently with protein expression, a significant increase of MGMT transcript was obtained with N6-BA, despite the compound sensitizing the cells to TMZ. In normal expressing T98 cells, only N6-BA was able to reduce MGMT mRNA levels (Figure 4B).

### IPA and N6-BA Affect DNA Methylation

**FIGURE 5:**
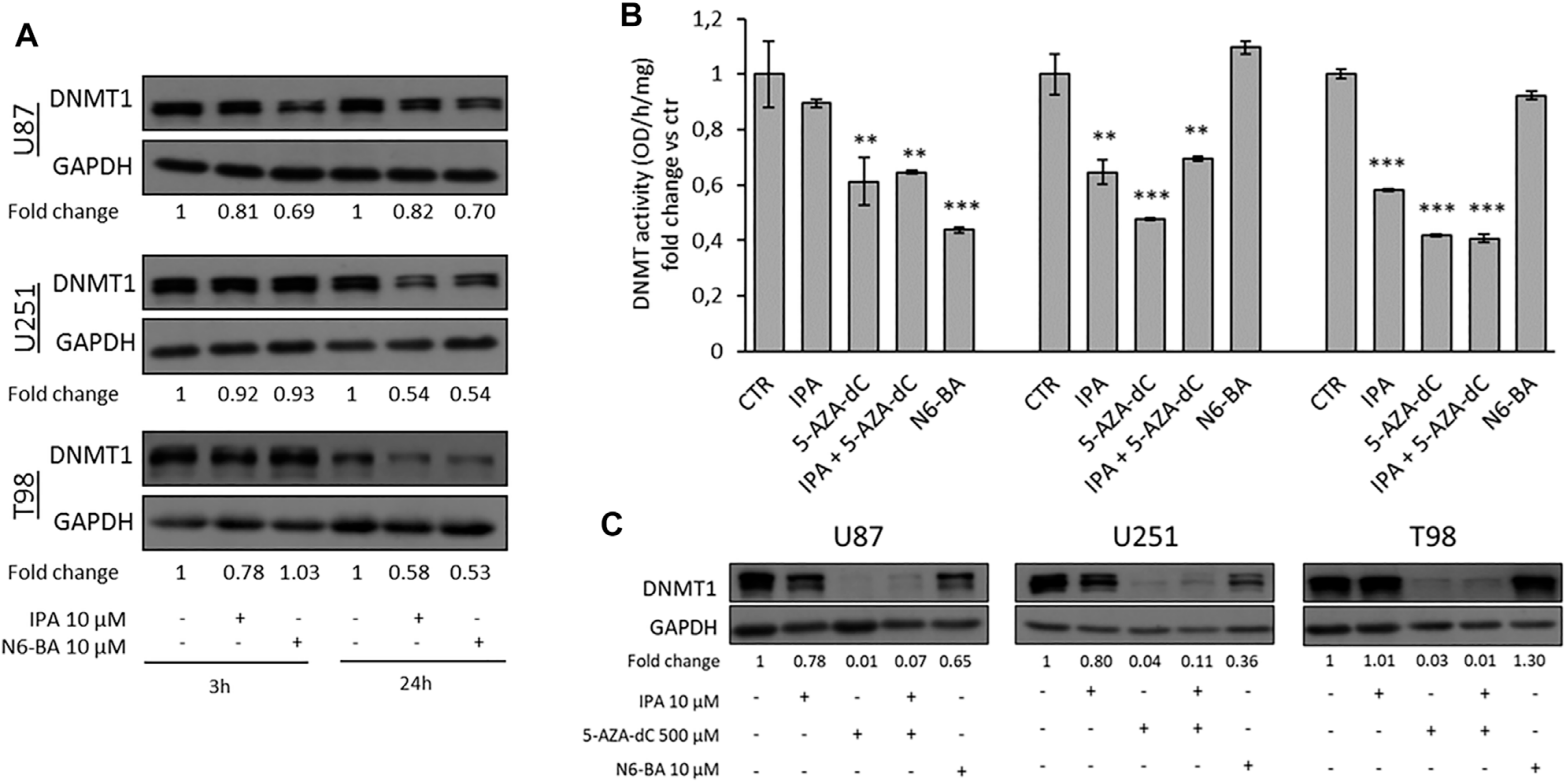
IPA and N6-BA affect DNMT expression and activity. **(A)** Representative western blot and relative densitometric analysis (fold change vs. control) of DNMT1 expression in U87, U251, and T98 cells treated with IPA or N6-BA. GAPDH was used as a loading control. **(B)** Colorimetric DNMT activity assay performed using nuclear extracts from GBM cells treated for 24 h with IPA (10 µM), 5-AZA-dC (500 µM), IPA+5-AZA-dC combination, or N6-BA (10 µM). DNMT activity (OD/h/mg) is expressed as fold change vs. control. Data are expressed as the mean ± SD of six independent experiments. **p < 0.01; ***p < 0.005 vs. control. **(C)** Representative western blot and relative densitometric analysis (fold change vs. control) of DNMT1 expression in extracts optimized to preserve the enzymatic activity of GBM treated for 24 h as indicated. GAPDH was used as a loading control.

Several studies in GBM revealed aberrant methylation patterns of specific genes, in addition to MGMT, and suggested that methylation profiles may be used to improve diagnostic accuracy and predict therapeutic responses (Wenger et al., 2019). The evidence that IPA and its analog N6-BA could influence the expression of methylated genes involved in tumor-related mechanisms, such as FBXW7, MGMT, and potentially others, together with the interference on TREs, suggests that they could exert their anti-cancer actions through transcriptional and epigenetic mechanisms. DNA methylation, occurring at C5-position of cytosine, is the major epigenetic modification responsible for chromatin dynamics, triggered and maintained by DNA methyltransferases (DNMTs) that use S-adenosylmethionine as a methyl donor (Laisné et al., 2018). To prove our hypothesis, we first evaluated the expression of DNMT1 (prevalently involved in methylation maintenance) in whole-cell extracts of GBM cell lines treated with IPA or N6-BA. Western blot analysis showed that, at short time, DNMT1 protein amount was effectively reduced by IPA and N6-BA in U87 but only by IPA in T98. After 24 h, both the molecules were able to reduce DNMT1 expression in the three GBM cell lines, with an equal extent in U251 and T98 (Figure 5A).

Subsequently, aimed to evaluate the ability of the two compounds to interfere with epigenetic regulation, we compared their effects with those of a known DNMT inhibitor, used as demethylating agent. Firstly, we performed a colorimetric assay to quantify DNMT activity in nuclear extracts of GBM cells treated for 24 h with IPA, N6-BA, 5-Aza-2′-deoxycytidine (5-AZA-dC or decitabine), or a combination of IPA and 5-Aza-dC. The analysis evidenced that, as expected, 5-AZA-dC treatment resulted in a significative DNMT activity inhibition. IPA was able to robustly reduce the enzymatic activity in U251 and T98 cell lines (about 40–45% of inhibition), while in U87 cells, despite the slight decrease, the effect did not reach statistical significance. Overall, the results suggested that IPA as a single agent behaves similarly to the known inhibitor but interestingly, when it was combined with 5-AZA-dC, it slightly restored DNMT activity in U251 and U87. The aza-cytosine substitutes cytosine during DNA replication and becomes substrates for DNMTs. The inhibition occurs because the enzymes covalently bond the modified cytosine but fail to transfer the methyl group, consequently undergoing degradation (Stresemann and Lyko, 2008). We speculated that IPA, as modified adenosine, used together with the 5-AZA-dC could weakly interfere with it for DNMT inhibition. Finally, a significant reduction following N6-BA treatment was only detected in U87 (Figure 5B). DNMT1 expression in nuclear extracts substantially confirmed the trend observed in whole-cell lysates, with some discrepancies probably due to the purification methods (Figure 5C).

**FIGURE 6:**
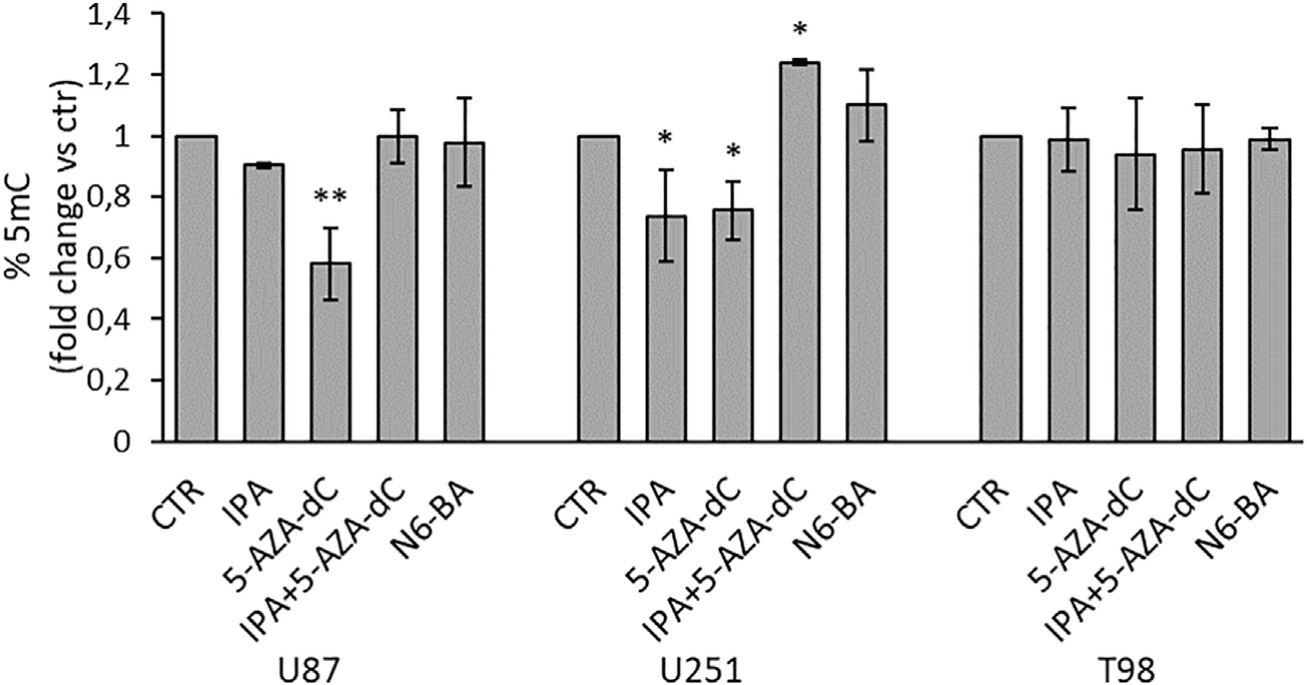
Effect on global DNA methylation in GBM cell lines. Quantification of global DNA methylation (ELISA) performed using 100 ng of genomic DNA isolated from GBM cells treated for 24 h with IPA (10 µM), 5-AZA-dC (500 µM), IPA+5-AZA-dC combination, or N6-BA (10 µM). DNA methylation (% 5-mC) was expressed as fold change vs. control. Data are expressed as the mean ± SD of three independent experiments. *p < 0.05; **p < 0.01 vs. control.

To strengthen these observations, we evaluated quantitative changes in global DNA methylation, measuring the percentage of 5-methylcytosine (% 5-mC). For this purpose, we isolated genomic DNA from GBM cell lines under the same experimental setting used for the DNMT enzymatic assay. In U87 and U251, consistent with previous data, 5-AZA-dC as a single agent reduced % 5-mC with a commensurate effect to the observed DNMT inhibition (Figure 6). Coherently with the effect on DNMT activity, IPA significantly decreased global methylation in U251 and slightly, although not significantly, in U87 cells, both harboring methylation of MGMT and FBXW7 promoters. Furthermore, the IPA/5-AZA-dC combination reverted the effect of 5-AZA-dC alone in U87 and even produced an increase of methylation reaching statistical significance in U251 (Figure 6). This could be explained with the evidence that high doses of DNA methylation inhibitors, like those used in clinical practice, cause a quick and transient increase of methylation in a small fraction of CpGs might reflect an adaptation response to the inhibitory stimulus, and hypomethylation occurs after several days (Giri and Aittokallio, 2019). Surprisingly, despite the inhibition of DNMT activity, no changes in %5 mC were observed in T98 (Figure 6). However, in this precise cell line, it was reported that DNMT suppression did not influence the methylation status in a subset of repressed genes displaying promoter hypermethylation but was instead associated with changes in histone modifications (Foltz et al., 2009).

Overall, these results collectively suggested that IPA, and to a less extent, N6-BA, could potentially modulate specific cancer-related genes through a complex mechanism that might involve DNA methyltransferase expression and/or activity and chromatin remodeling.

### IPA and N6-BA Influence Histone Modifications

Despite the inhibition of DNMT activity and global DNA methylation mediated by the compounds, coherent with FBXW7 restoration in U87 and U251, it might seem counterintuitive if we consider the result of the luciferase assay.

**FIGURE 7:**
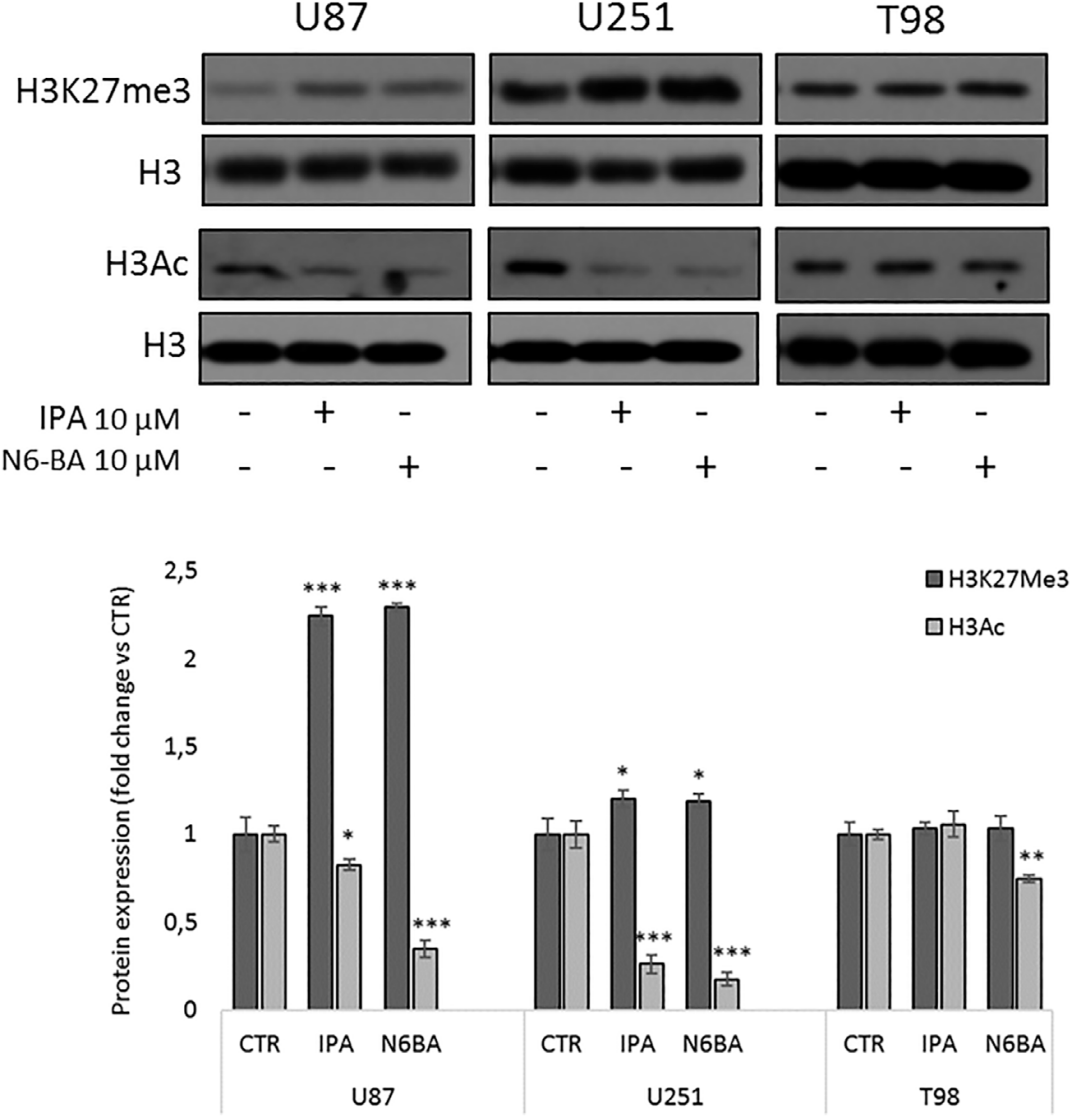
IPA and N6-BA influence histone marks associated with gene repression. Representative western blot and densitometric analysis of H3K27me3 and H3Ac in purified histone fractions of GBM cell lines treated with IPA or N6-BA after 24 h. H3 was used as a loading control. The histograms report the quantification of bands intensity expressed as mean ± SD of three independent experiments. *p < 0.05, **p <0.01, and ***p < 0.005 vs. control.

To further understand the involvement of epigenetic mechanisms in the antitumor effect of the two compounds, we analyzed their effect on histone marks commonly related to gene repression or active transcription. In U87 and U251, after 24 h, both IPA and N6-BA significantly increase the trimethylation on lysine 27 of histone H3 (H3K27me3) (Figure 7). Coherently, a reduction of global lysine acetylation (H3 pan-acetyl) was observed in all cell lines analyzed (Figure 7). In T98 cells, the treatments failed to modulate H3K27me3, and pan-lysine acetylation of H3 was only reduced by N6-BA (Figure 7). Overall, the global trend of examined histone marks suggested that IPA and N6-BA induced a transcriptional repressive trend, which is not aligned with enzymatic inhibition of DNMT but is compatible with MGMT repression and the observed downregulation of FBXW7-targets playing crucial roles in cell metabolism and survival (c-Myc, SREBP, and Mcl1).

### Effects of IPA and N6-BA in Glioma Primary Cell Lines

**FIGURE 8:**
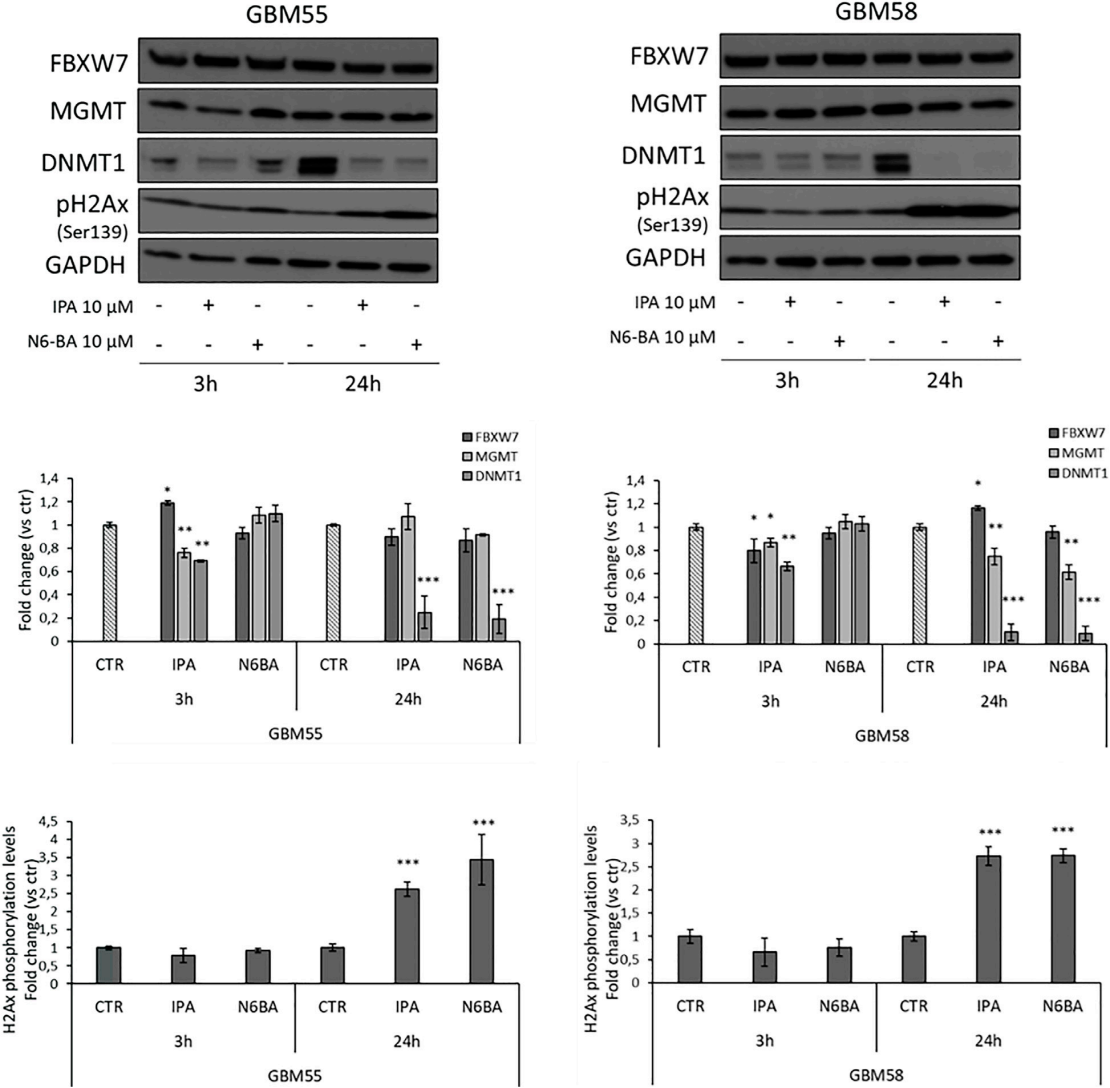
IPA and N6-BA antitumor effects in GBM primary cell lines. Representative western blot and densitometric analysis of FBXW7, DNMT1, and MGMT expression and H2Ax phosphorylation levels in GBM primary cell lines treated for 3 or 24 h with the indicated molecules. GAPDH was used as a loading control. The histograms report the quantification of bands intensity expressed as mean ± SD of three independent experiments. *p < 0.05, **p <0.01, and ***p < 0.005 vs. control.

The overall evidence emerging from this dissertation is that IPA or its analog N6-BA modulates different cancer-related effectors plausibly interfering upstream with their regulation. This is particularly intriguing in GBM, whose intricate epigenetic landscape further complicates its treatment and management. Aimed to further investigate the antitumor potential of the adenosine compounds in GBM models endowed with unique genetic features, we established two primary cell lines from some fresh glioma patients’ resected tumors. Through western blot analysis, we evaluated IPA or N6-BA effects on selected targets in primary cell lines verifying that IPA produced a slight but significant upregulation of FBXW7 after 3 h in GBM55 and 24 h in GBM58. Moreover, IPA was also able to repress MGMT levels, both at early and prolonged time in GBM58 but only after 3 h in GBM55. Finally, IPA mediated an early decrease of DNMT1 levels, followed by a strong reduction or even an abrogation after 24 h in both primary cell lines. Very similar to IPA, the benzyl analog N6-BA substantially decreased DNMT1 and MGMT expression after 24 h but failed to significantly modulate FBXW7 (Figure 8). Expression changes at 24h were accompanied by a significant increase of H2Ax phosphorylation levels (Ser139), a mark of DNA damage (Figure 8). These preliminary observations in GBM primary cell lines enforce the hypothesis that IPA and N6-BA could interfere with DNA remodeling, and they could represent new valid therapeutic tools in GBM.

## Discussion

Although the understanding of the molecular mechanisms that drive gliomagenesis has made great progress, GBM remains one of the most difficult cancers to treat. Surgery and radio- or chemotherapy, mainly with TMZ, significantly improved patient survival in most cases. The ability of TMZ to effectively cross the blood–brain barrier and reach therapeutic concentrations in the brain makes it the choice compound in GBM management. However, high toxicity and acquired resistance are very common and often related to tumor recurrence, limiting the success of actual intervention strategies (Goenka et al., 2021). Hence, the identification and development of second-line treatment for GBM patients are dramatically necessary.

The results shown here confirm our previous finding in CRC (Fiore et al., 2019), highlighting that even in GBM, IPA and its synthetic analog N6-BA upregulate the expression and, indirectly, the activity of the oncosuppressor FBXW7 emerged in the last years as an appealing therapeutic target in different cancers. In GBM, FBXW7 is involved in DNA damage response (Humphreys et al., 2021). The WD40 domain of F-box proteins, which is responsible for substrate binding, is also required to bind the poly (ADP-ribose) (PAR) immediately following DNA double-strand breaks (DSBs) and mediates rapid FBXW7 recruitment for X-ray repair cross-complementing protein 4 (XRCC4) ubiquitination and activation of non-homologous end-joining (NHEJ) repair system (Zhang et al., 2019). In a recent study, FBXW7 was related to TMZ resistance and, in particular, FBXW7 re-expression in a TMZ-resistant U251 clone partially sensitized to the drug, while FBXW7 silencing produced the opposite effect (Lin et al., 2018). In U251 and GBM primary cell lines, the evidence that IPA amplifies DNA damage before irradiation (Navarra et al., 2020), together with the finding of FBXW7 upregulation, here debated, are extremely intriguing since GBM therapeutic protocols include different combinations of ionizing radiation and TMZ, and the resistance is modulated by DNA repair systems (Stupp et al., 2005; Oldrini et al., 2020).

We showed that IPA pre-treatment can boost the killing effect of TMZ, starting from a concentration range much lower than TMZ EC50, in GBM cell lines already sensitive to the alkylating agent (Lee, 2016), where the modified adenosines strongly impact on proliferation and increase FBXW7 expression. In T98 cells, poorly responsive to TMZ, IPA and N6-BA did not substantially alter FBXW7 protein levels, but both compounds reduced MGMT levels, whose expression, epigenetically suppressed in U87 and U251, predicts therapeutic susceptibility to TMZ. Despite this, IPA pre-treatment was not able to significantly improve TMZ efficacy in T98, at least at the dose and time points analyzed. However, in this resistant model, cell death is induced by higher TMZ concentration and increasing incubation time. Moreover, the acquisition of TMZ resistance *in vitro* is a complex mechanism that involves rapid and is not reversible epigenetic rearrangements, thus complicating the individuation of the precise timeframe in which cells are tolerant to the drug (Rabé et al., 2020).

Taking into account the relevance of the epigenetic landscape in gliomas and, in particular, in the prediction of treatment sensitivity, in recent years, pharmacological research is increasingly considering the possibility of including epidrugs in therapeutic options for GBM, with particular regard to personalized therapy (Zang et al., 2018; Phillips et al., 2020). Several clinical trials are currently evaluating the effect of different epigenetic modulators, including DNA-methyltransferase inhibitors (DNMTi), used alone or in combined therapies with TMZ or radiations (ClinicalTrials.gov, 2021; Wu et al., 2021), and various studies have proposed epidrugs and DNMTi, among other, to optimize the use of TMZ for personalized medicine in GBM (Seelan et al., 2018; Yamashita et al., 2019; Belter et al., 2020; Gallitto et al., 2020; Moon et al., 2020; Sun and Turcan, 2021).

The main evidence emerging from this study is that IPA and its analog N6-BA could influence the expression of methylated genes involved in tumor-related mechanisms, such as MGMT, FBXW7, and potentially others, suggesting the existence of an epigenetic regulation behind their pleiotropic actions. Our investigations revealed that the modified adenosines overall behave similarly to 5-AZA-dC, known DNMTi, affecting both the expression and activity of DNMT1,a crucial enzyme in the maintenance of DNA methylation patterns. However, global DNA methylation did not completely reflect DNMT activity inhibition trend and, in particular, we observed different IPA behaviors. In U87 and U251 cell lines, IPA reduces global methylation when used as a single agent, but in combination with 5-AZA-dC increases the total percentage of 5-methylcytosine. This evidence is suggestive of an adaptive response to an excessive inhibitory stimulus, as explained in a recent report analyzing DNMTi effects on colon cancer genome methylation (Giri and Aittokallio, 2019). On the other hand, the slight reversion of 5-AZA-dC-mediated inhibition of DNMTs, observed in U87 and U251 cell lines after combined treatment with IPA, lead us to speculate that an interference between the two compounds occurs, probably ascribable to a sort of competition. Since we have not demonstrated the direct interaction of IPA or N6-BA with DNMT enzymes, it is also possible that an indirect interference with DNA enzyme dynamics could result from direct interaction with the DNA double helix. In fact, previous evidence indicates that, in an aqueous solution, IPA-DNA interaction occurs at the DNA surface (Rajabi et al., 2010). Moreover, recent evidence clarified the dynamic of endogenous IPA metabolism. CDK5RAP1 (Cdk5 regulatory subunit-associated protein 1) detoxifies IPA by conversion into ms2i6A (2-Methylthio-N6-isopentenyladenosine). Since ms2i6A is an evolutionarily conserved modification, influencing the functions of the transcription machinery (Yamamoto et al., 2019), we cannot exclude that exogenous free IPA disturbs DNA and RNA modification processes and/or integrates into both DNA and RNA. Additionally, it is also plausible that IPA or its metabolites interfere with other methyltransferases that exploit S-adenosylmethionine as a methyl donor.

FBXW7 restoring in CRC or GBM cancer cells harboring promoter methylation is consistent with DNMT inhibition mediated by the modified adenosines. In 2009, Colombo et al., reported that in lung and breast cancer cells, IPA affects gene expression of a subset of genes, mainly involved in cell cycle control, apoptosis, transcriptional regulation and protein modification. Of about one hundred genes, most were upregulated, and only three were downregulated (Colombo et al., 2009), enforcing our data on the IPA-mediated DNMT inhibition.

It is worth mentioning that non-specific DNMT inhibition, like that occurring after treatment with 5-AZA-dC, often produces an intricate flat effect due to non-specific reorganization of DNA. The process is orchestrated by a multitude of actors, in addition to DNMTs and differently expressed in tumors, including 5-methylcytosine hydroxylases (TETs), histone modification enzymes (HACs, HDACs, HMTs, and KDMs), ubiquitin-like proteins that recognize methylated CpG sites or histones, and last but not least non-coding RNA (Cheng et al., 2019). This aspect could explain some counterintuitive results, like those from luciferase assay and the analysis of global histone modifications, that seem unaligned with DNMT inhibition. Coherently with promoter inactivation on Myc, NFkB, HIF-1α, or Elk-SRF responsive elements observed in U87 cells treated with IPA, analysis of histones modification suggests a repressive global trend, with the increase of H3K27Me3 and a clear decrease of H3Ac. Taking into account the effect on DNMT activity, these data are unexpected but plausible since the reduction of luciferase activity on the TREs analyzed could be linked with the FBXW7-mediated degradation of Myc, NFkB, or HIF-1α substrates, induced by IPA.

Interestingly, the inhibition of DNMTs after treatment with 5-AZA-dC produces a significant reduction of HDAC4 and HDAC5 promoter methylation, with a consequent increase in their gene expression, in U87 and U251 cells, but not in the T98 cell line (Gomes et al., 2011). This evidence is consistent with our data and suggests that the reduction of H3Ac observed in IPA-treated U87 and U251 cells, despite not being expected, could be ascribable to the restoration of some HDACs expression and consequent reduction in global histone acetylation. Moreover, in the last years, it became evident that, beyond promoters, gene body methylation also plays a fundamental role in regulating gene expression, and in GBM, this process takes a role in chemoresistance (Moen et al., 2014; Yang et al., 2014). It has been found that 5-AZA-dC is able to reactivate gene expression and also to demethylate DNA in gene bodies (Yang et al., 2014; Seelan et al., 2018) and this occurrence results in mitigation of overexpressed oncogenes. The downregulation of the oncogenes seems related to a transient rapid remethylation after drug treatment that involves DNMT3B and is associated with increased chromatin accessibility and a rise in H3K27Me3, the polycomb mark that regulates remethylation kinetics. Many of these downregulated genes are linked to cancer metabolic pathways and are regulated by c-Myc (Yang et al., 2014). Of note, the interplay between the mevalonate pathway and c-Myc regulation has been previously described (Wang et al., 2017).

A possible account for IPA-mediated downregulation of the MGMT expression could stem from gene body methylation. Moen et al., demonstrated that demethylation of MGMT gene body, induced by 5-AZA-dC treatment, decreases the expression of MGMT and then the pre-treatment with the DNMTi sensitize the cells to TMZ (Moen et al., 2014). In our models, the reduction of MGMT mRNA expression has been verified only in U87 and U251, where IPA negatively modulates global DNA methylation but not in T98 cells. In this work, we did not demonstrate the specific inhibition of DNA methylation on specific regions, but we can speculate that this counteractive result could be explained with a possible effect on the MGMT gene body. Furthermore, it has been reported that preconditioning with DNMTi sensitizes GBM cells to TMZ through a mechanism that involves re-expression of MMR (DNA mismatch repair) proteins, like MLH1 (MutL homolog 1), enhancing DSB. MLH1 upregulation induced by decitabine is probably not due to promoter demethylation but instead an indirect mechanism that induces E2F1 binding in the MLH1 promoter region (Gallitto et al., 2020).

Overall, our results suggest that the modified adenosine, and mainly the lead compound IPA, behave like DNMTi. This preliminary evidence, for the first time, opens the possibility of considering this class of molecules as epigenetic tools to overcome resistance or enhance chemo- and radiotherapy efficacy, mainly when combined with TMZ, in glioblastoma and other cancers.

Beyond the lack of *in vivo* studies, the weakness of this work is that the *in vitro* assays used here evaluated a global effect on DNA methylation and did not discriminate between *de novo* or maintenance DNMT activity. Of course, future investigations are needed to clarify if the effects are selective to specific DNMTs, and genome-wide DNA methylation analysis will help to understand and extensively dissect the dynamic of DNA modification triggered by the molecules.

## Data Availability

The raw data supporting the conclusion of this article will be made available by the authors, without undue reservation.
