# Supplementary material for: Modified Adenosines Sensitize Glioblastoma Cells to Temozolomide by Affecting DNA Methyltransferases
Source: Front Pharmacol. 2022 Apr 26;13:815646. doi: 10.3389/fphar.2022.815646 (PMC9086827; doi:10.3389/fphar.2022.815646)
Supplement: Supplementary file 1 [file DataSheet1.docx]

Supplementary Material

## Supplementary Figures


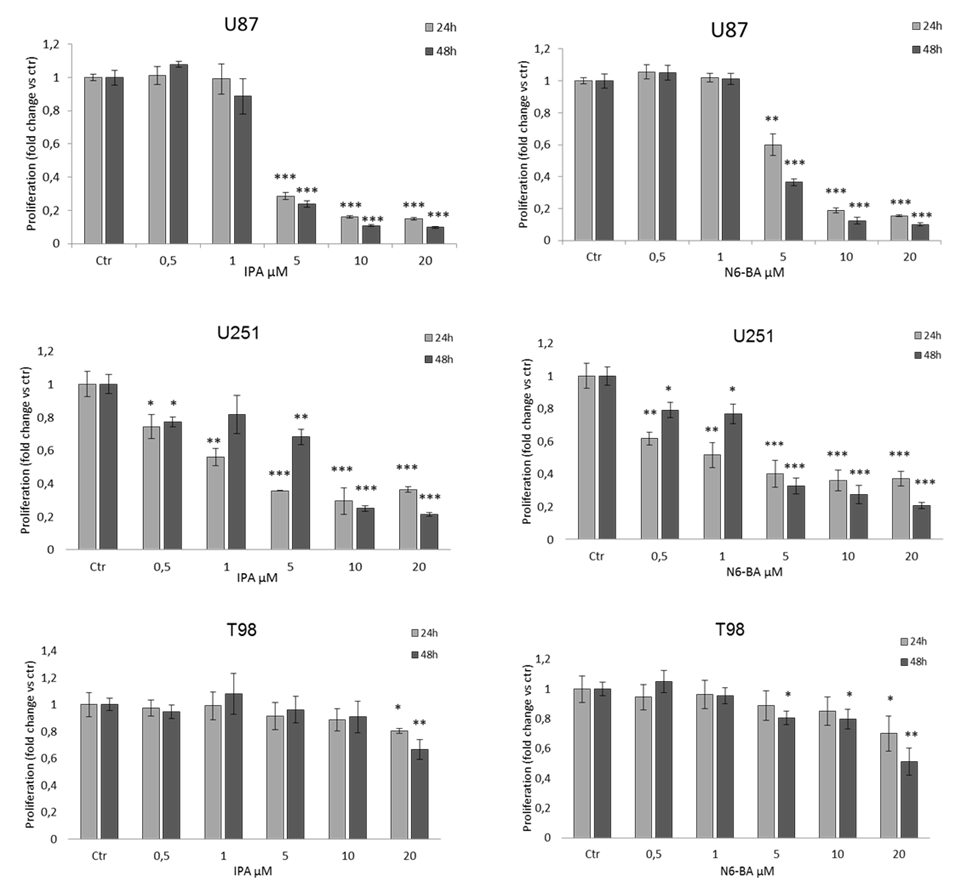


**Supplementary Figure 1.** Effect of IPA and N6-BA on GBM cells *in vitro*. BrdU incorporation assay in U87, U251 and T98 cells treated with indicated concentrations of IPA (left column) or N6-BA (right column), for 24 h or 48 h. Data are expressed as mean ± SD of at least three independent experiments. * p < 0.05, ** p < 0.01, *** p < 0.005 vs. control.


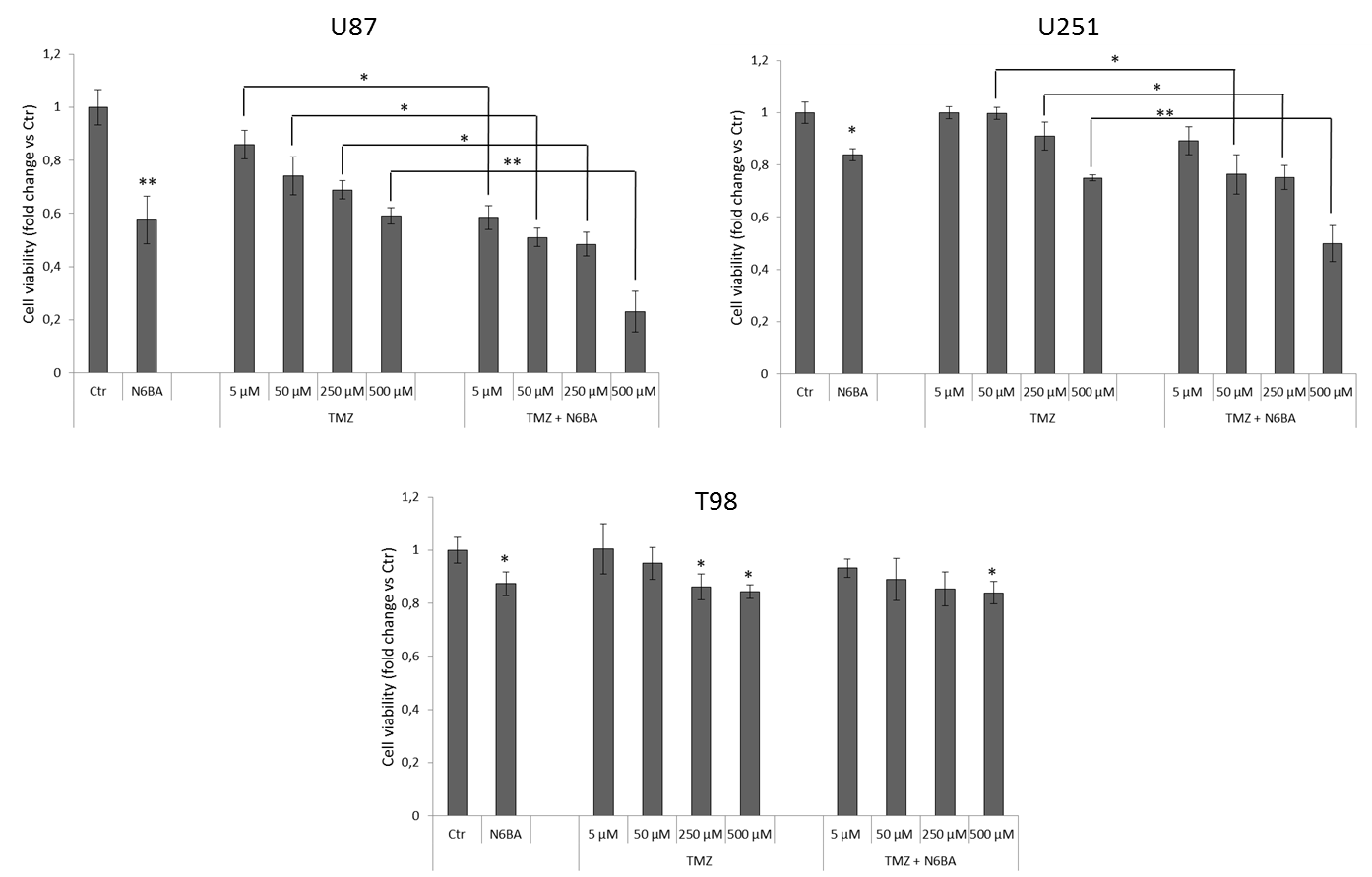


**Supplementary figure 2.** N6-BA sensitize GBM to TMZ. GBM cells were exposed to 10 µM N6-BA or vehicle (DMSO) for 24h. After the incubation, medium was removed and replaced with fresh one containing the indicated concentrations of TMZ or the relative vehicle alone. MTT assay was used to assess cell viability after 72h, expressed as the mean ± SD of three independent experiments. * p < 0,05 ** p < 0.01, in U87 and U251, *p<0.05 vs control in T98
